# Prevalence, management and burden of episodic and chronic headaches—a cross-sectional multicentre study in eight Austrian headache centres

**DOI:** 10.1186/s10194-015-0531-7

**Published:** 2015-05-19

**Authors:** Karin Zebenholzer, Colette Andree, Anita Lechner, Gregor Broessner, Christian Lampl, Gernot Luthringshausen, Albert Wuschitz, Sonja-Maria Obmann, Klaus Berek, Christian Wöber

**Affiliations:** Department of Neurology, Medical University of Vienna, Vienna, Austria; CRP Santé, Strassen, Luxemburg; University Basle Department of Pharmaceutical Sciences, Basel, Switzerland; Department of Neurology, Medical University of Graz, Graz, Austria; Department of Neurology, Medical University of Innsbruck, Innsbruck, Austria; Headache Centre Seilerstätte, Hospital Sisters of Charity Linz, Linz, Austria; University Clinic of Neurology, Christian-Doppler-Klinik Salzburg, Salzburg, Austria; Neurological Office Vienna, Vienna, Austria; Department of Neurology, Klinikum Klagenfurt, Klagenfurt, Austria; Department of Neurology, a.ö. KH Kufstein, Kufstein, Austria

**Keywords:** Episodic headache, Chronic headache, Migraine, Medication overuse headache, Prophylactic treatment, Health care service, Quality of life, Burden, Anxiety, Depression

## Abstract

**Background:**

Episodic and chronic headaches (EH, CH) are highly prevalent disorders. Severely affected patients are usually referred to headache centres. In Austria, at least one headache centre is available in seven of nine states, but detailed multicentre data are missing. Therefore we studied prevalence rates, use of medication and health care services, impact of headaches, and comorbid depression and anxiety.

**Methods:**

We included consecutive patients from eight Austrian outpatient headache centres. The patients filled-in the Eurolight questionnaire. In addition, the treating neurologist completed a questionnaire on clinical diagnoses and ever-before prophylactic medications.

**Results:**

Of 598 patients screened, 441 questionnaires were analysed (79 % female, mean age 41.1 years). According to the Eurolight algorithm, 56.4 % of the patients had EH, 38.3 % had CH and 5.2 % did not give their headache frequency. The prevalence rates of migraine, tension-type headache, and probable medication overuse headache (pMOH) were 48.5 %, 6.3 % and 15.9 %, respectively. The concordance between clinical and Eurolight diagnoses was good for EH and moderate for CH. During the preceding month, acute medication was used by 90.9 % of the patients and prophylactic medication by 34 %. Ever-before use of five standard prophylactic drugs was recorded in 52.3 %. The proportion of patients with current pharmacoprophylaxis did not differ in EH and CH, whereas ever-before use was more common in CH (62.5 % was 45,3 %, *p* = 0.02). Patients with CH significantly more often consulted general practitioners and emergency departments, had a lower quality of life and more often signs of depression and anxiety.

**Conclusion:**

This study provides comprehensive data from eight Austrian headache centres for the first time. We found a substantial number of patients with CH including pMOH and its association with more common utilization of health care facilities and greater burden. The low use of prophylactic medication requires further examination.

## Background

Headache is one of the most frequent neurological disorders interfering with everyday life. The one-year prevalence is 10–18 % in migraine, and 31–90 % in tension-type headache (TTH) [1–3]. For Austria the one-year prevalence of migraine was 10 % [4]. In a European survey migraine was found in 36 %, probable medication overuse headache (pMOH) in 3 % and any chronic headache in 7.6 % of the participants during the preceding year. These high prevalence rates were attributed to the study design [5]. Headaches, especially chronic headache and medication overuse headache (MOH), are associated with comorbidities such as depression, anxiety disorders, restless legs syndrome [6–8] and reduced quality of life [9]. The impact on the patients’ lives is high with regard to lost workdays, and lost family, social or leisure activities [5]. MOH, with a life-time prevalence of 1–2 % [1, 5], often represents a major challenge, exceeding the impact of migraine or TTH on everyday’s life [5]. In specialized headache centres, patients with medication overuse can account for 30–45 % of all patients [10–12]. In Austria, at least one headache centre is available in seven of nine states. Data on MOH are only available from one centre, i.e. the Department of Neurology at the Medical University of Vienna, performing in-patient withdrawal treatment in 40–50 patients every year [13–15]. Detailed multicentre data on MOH and other frequent headaches, however, are missing. Therefore, we performed a prospective study in eight Austrian headache centres and evaluated (i) the prevalence of episodic (EH) and chronic headaches (CH), (ii) the prevalence of migraine, TTH and pMOH, (iii) the use of acute and prophylactic medication, (iv) the use of health care services, (v) the burden of headache and, (vi) comorbid depression and anxiety.

## Methods

In April 2011 and September 2011 all consecutive patients attending one of eight Austrian headache centres for a first time visit or a follow-up visit were invited to participate in the study. Exclusion criteria were secondary headache except medication overuse headache, fibromyalgia, other chronic pain disorders, and lacking knowledge of German. Four centres were at departments of medical universities (Graz, Innsbruck, Salzburg, Vienna), three centres were at large hospitals (Klagenfurt, Kufstein, Linz) and one centre was a large neurological office in Vienna. The study was approved by the ethics committees of the Medical Universities of Vienna, Graz, Innsbruck and Salzburg, and local ethics committees of the other participating departments.

After giving written informed consent the patients filled in the Eurolight questionnaire covering biographic data, headache symptoms, use of acute and prophylactic medication, former examinations due to headaches, quality of life, symptoms of anxiety and depression, the impact of headache and health related quality of life [16]. The Eurolight questionnaire differentiates EH (headache on < 15 days/month) from CH (headache on ≥ 15 days/month) and is validated for diagnosing migraine, probable migraine, TTH, probable TTH, and pMOH according to the International Classification of Headache Disorders, 2^nd^ edition (ICHD-2) [17] and for assessing the impact of headache disorders [16]. Probable MOH was diagnosed when headaches occurred on ≥ 15 days per month, lasted ≥ 4 h, and the frequency of acute medication use was ≥ 15 days/month for simple analgesics and ≥ 10 days/month for compound analgesics, opioids, triptans, or ergots [5]. The questionnaire does not allow diagnosing chronic migraine or chronic tension-type headache in the strict sense of ICHD-2 or its appendix. Therefore we separated two groups by their headache frequency as given in the questionnaire: patients with EH and patients with CH. The Eurolight questionnaire also includes the HALT index [18], WHOQoL-8 [19], and the Hospital Anxiety and Depression Scale (HADS) [20]. The HALT index captures in five questions the days lost completely or partially because of headache in the preceding three months and covers professional work, household activities or chores, and family, social or leisure activities [18]. To estimate the burden of headache, and because the data were not normally distributed, we summarized these lost days during the previous three months and categorized the impact of headache into 0–5 days lost, 6–10 days lost, 11–20 days lost, and >20 days lost. The WHOQoL-8 produces scores for domains related to quality of life (physical health, psychological, social relationships, environment) and it includes one facet on overall quality of life and general health [19]. The HADS is a screening instrument for depression and anxiety in patients with physical complaints. It is a self-rating questionnaire with two subscales comprising seven items each [20].

The treating neurologist filled in an additional questionnaire covering the clinical headache diagnosis and ever-before intake of five standard prophylactic medications, i.e. betablockers, flunarizine, valproate, topiramate and amitriptyline. For each of these drugs the treating neurologist had to assess, if it was contraindicated and if it ever was taken by the patient. In the latter case, four additional questions had to be answered: Was the treatment stopped because of intolerable adverse effects? Was the taken dose sufficient [21]? Was treatment continued for at least three months? Did the treatment result in a decrease of headache frequency by at least 50 %? Each of these questions was answered “yes”, “no” or “unknown” based on information from the patient and from medical records.

### Statistics

We applied the standard computerized algorithm to analyse data derived from the Eurolight questionnaire [22]. We used numbers and percentages for descriptive statistics and calculated Chi^2^ tests for comparing patients with EH and CH. Two-sided p-values < 0.05 were considered as statistically significant. For evaluating the concordance between clinical diagnoses and Eurolight diagnoses we defined moderate, good and excellent concordance as agreement of diagnoses in 40–60 %, 61–80 %, and ≥ 81 % respectively. Statistical analyses were performed using SAS version 9.2 and SPSS 20.0.

## Results

We invited 598 consecutive patients, 121 denied participation or had to be excluded. Main reasons for exclusion were lacking fluency in German, particularly at the Medical University of Vienna. Thirty-six questionnaires had to be excluded because of incomplete data. Therefore the final analysis was based on 441 Eurolight questionnaires. The dropout rates per centre ranged from 2 to 14 %. We included 111 patients at the department of neurology in Vienna, 76 in Graz, 69 in the neurological practice in Vienna, 51 in Kufstein, 48 in Klagenfurt, 47 in Linz, 24 in Salzburg and 15 in Innsbruck. In addition completed questionnaires on clinical diagnoses and prophylactic medications were available from 392 patients.

### Biographic findings

Ninety-three patients (21 %) were male and 348 (79 %) were female. The proportion of female patients ranged from 64.6 to 87.5 % in the eight study centres. The patients’ mean age was 41.1 ± 14.0 years (16–79) with a range of 32.4–45.6 years (mean) in the eight centres. The patients’ occupational status is shown in Table 1.

**Table 1:** Occupation and income, comparison between patients with EH and CH

	EH *n* = 249		CH *n* = 169		Chi^2^	*p*
	n	%	n	%		
Occupation, missing data: 23						
Employee	168	70.6	79	49.4		
Housewife/househusband	5	2.1	13	8.1		
Student	28	11.8	17	10.6		
Unemployed	11	4.6	24	15.0		
Retired	26	10.9	27	16.9	29.0	<0.0001
Income, missing data: 49						
<1500 Euro/month	105	44.9	103	65.2		
1500–3500 Euro/month	115	49.1	48	30.4		
>3500 Euro/month	14	6.0	7	4.4	17.2	0.018

EH, Episodic headache, CH, Chronic headache

### Headache chronicity

Two-hundred-forty-nine patients (56.5 %) had EH and 169 (38.3 %) patients had CH, in 23 the patients’ frequency data were missing. The prevalence of CH showed a wide range of 17.2–56.8 % in the eight headache centres, with the lowest rate in the neurological office and the highest in Klagenfurt. Age did not differ in patients with EH and CH, whereas the percentage of males was higher in CH than in EH (30 % vs. 14.7 %; Chi2 = 13.5, *p* < 0.001).

### Headache diagnoses

Among all patients, 214 (48.5 %) had migraine (thereof 85 probable migraine), 28 (6.3 %) had TTH (thereof 10 probable TTH), 70 (15.9 %) had pMOH, 99 (22.4 %) had CH not further specified by the Eurolight algorithm, and 7 (1.6 %) had other headaches. When the diagnosis CH or pMOH was made, or the patients did not give their headache frequency, the Eurolight algorithm did not allow differentiating between migraine, TTH and other headaches. However, all patients had to give the characteristics of their most bothersome headache type. Accordingly some differentiation is possible and summarized in Table 2.

**Table 2:** Comparison of headache diagnoses in patients with chronic headache and patients with missing headache frequency based on the “worst headache” in the Eurolight questionnaire

HA diagnosis (worst HA)	CH		CH with pMOH		CH without pMOH		HA frequency missing	
	*n* = 169		*n* = 70		*n* = 99		*n* = 23	
	n	%	n	%	n	%	N	%
Migrainous HA	137	81.1	66	94.3	71	71.7	16	69.6
Tension-type like HA	27	16	3	4.3	24	24.2	5	21.7
Other HA	5	3	1	1.4	4	4	2	8.7

HA, Headache, CH, Chronic headache, pMOH, Probable medication overuse headache

The clinical diagnosis was concordant with the Eurolight diagnosis in 181 patients (78 %) with EH and in 102 patients (63.7 %) with CH. In the majority of patients with conflicting diagnoses the Eurolight diagnoses were probable migraine, probable TTH or other headaches.

### Medication

According to the Eurolight questionnaire most patients (90.9 %) used acute medication to treat their headache attacks during the past month. The proportion was significantly lower in CH than in EH (86.9 % vs. 94.4 %; Chi2 7.1; *p* = 0.008). Only 34 % of the patients reported any prophylactic medication at the time of the survey and there was no statistically significant difference between EH and CH.

**Fig. 1:**
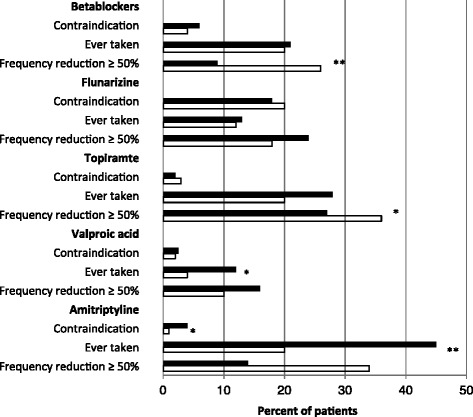
Percentages of patients with contraindications against, ever-before use of, and frequency reduction by ≥ 50 % by five standard prophylactic drugs for migraine and/or TTH in EH compared to CH, White bars = episodic headache, black bars = chronic headache, *p < 0.05; **p < 0.001

According to the additional questionnaire completed by the treating neurologists and covering betablockers, flunarizine, topiramate, valproic acid and amitriptyline, amitriptyline was previously or currently used most frequently (30.4 % of the patients) and valproic acid was used least frequently (7.4 % of the patients). In total, 52.3 % of the patients had ever used any prophylactic drug: 31.6 % had used one drug, 10.5 % two drugs and 10.2 % three or more. Significantly fewer patients with EH had ever-before used prophylactic drugs than patients with CH (45.3 % vs. 62.5 %; Chi^2^ = 12.3, *p* = 0.02). Fig. 1 shows details of prophylactic drug use: Significantly more patients with CH had ever used amitriptyline and valproic acid than patients with EH. Except for flunarizine (19.4 %) the rate of contraindications was below 10 %. Significantly more patients with EH using betablockers or topiramate had a frequency reduction of at least 50 % (Chi2 = 9.9, *p* = 0.0019; Chi2 = 7.0, *p* = 0.046). Using a sufficient dose [21] was most common for flunarizine (89.8 %) and least common for betablockers (72.5 %). More than 60 % of patients that were prescribed any of these prophylactic medications kept on taking them for more than three months.

### Use of health care services

Table 3 shows the use of health care services due to headache. Remarkably, the proportion of patients who had sought help in an emergency department was only 4.4 % in patients from the neurological office, but 13.3–19.2 % in patients from hospital based headache centres. Although patient numbers were relatively small for comparisons, patients with CH consulted a general practitioner or emergency department significantly more often than patients with EH. Magnetic resonance imaging (MRI) or computed tomography (CT) of the brain was done in 29.5 % of the patients with CH and in 32.7 % of the patients with EH during the previous year.

**Table 3:** Differences in the utilization of health care services between patients with episodic headache and chronic headache

Health care services	All patients		EH		CH		Chi^2^	P
Frequency data missing 23	n	%	n	%	n	%		
General practitioner	146	33.1	72	30.0	66	40.5	4.7	0.029
Neurologist	286	64.9	165	69.3	109	66.5	0.37	n.s.
Physical therapist	99	22.5	40	17.1	54	32.7	0.27	n.s.
Emergency department	63	14.3	34	14.7	27	16.6	13.1	0.0003

### Burden of headache, comorbidity

About one third of the patients (34.3 %) reported a negative impact of their headache on their career and 21.5 % indicated that their headaches had negative impact on their earnings. Only 49.9 % of the patients had the impression that their colleagues at work accepted their headaches. Significantly more patients with CH than with EH were unemployed or retired, and patients with CH had significantly lower incomes (Table 1).

Table 4 shows the cumulative number of lost days according to the HALT index. Patients with CH had a significantly higher burden. This categorization does not yield intuitive information about the amount of time actually lost. But the range of lost days was very wide (0–90), and therefore mean values or medians do not reflect the true loss satisfactorily, either. Only few patients accounted for a disproportional part of the reported burden; 26 (5.9 %) patients reported ≥ 45 lost days in at least one of lost work, housework or social days. The patients’ health-related quality of life, anxious and depressive state are shown in Table 4. Patients with CH had significantly more symptoms of anxiety and depression than patients with EH. Quality of life was significantly worse in patients with CH than in patients with EH. Table 5 gives quality of life, depression and anxiety in patients with CH with and without pMOH and shows no statistically significant differences.

**Table 4:** Quality of life, anxiety, depression and burden of headache in all patients and in patients with episodic and chronic headache

	All patients		EH		CH			
	n	%	n	%	n	%	Chi^2^	p
Quality of life							38.2	<0.0001
Missing data 28								
Very poor	18	4.2	3	1.2	13	7.9		
Poor	34	7.8	11	4.4	20	12.2		
Neither good nor poor	100	23.0	44	17.7	50	30.5		
Good	199	45.9	137	55.0	55	33.6		
Very good	83	19.1	54	21.7	26	15.9		
HADS Anxiety							19.6	<0.0001
Missing data 37								
No anxiety	241	54.6	160	65.6	74	46.3		
Possible anxiety	79	17.9	45	18.4	32	20.0		
Probable anxiety	102	23.1	39	16.0	54	33.8		
HADS Depression							39.0	<0.0001
Missing data 45								
No depression	281	63.7	186	77.8	86	54.8		
Possible depression	76	17.2	41	17.2	30	19.1		
Probable depression	58	13.3	12	5.0	41	26.1		
HALT Index								
Missing data 54								
0–5 days lost	96	24.8	67	29.4	25	17.6	32	<0.001
6–10 days lost	50	12.9	38	17.7	9	6.3		
11–20 days lost	66	17.1	46	20.2	18	12.7		
>20 days lost	175	45.2	77	33.8	90	63.4

**Table 5:** Quality of life, anxiety and depression in patients with chronic headache with and without probable medication overuse headache

	CH with pMOH		CH without pMOH			
	n	%	n	%	Chi^2^	p
Quality of life					3.0	n.s.
Missing data	3		2			
Very poor	7	10,4	6	6,2		
Poor	9	13,4	11	11,3		
Neither good nor poor	21	31,3	29	29,9		
Good	22	32,8	33	34,0		
Very good	8	11,9	18	18,6		
HADS Anxiety					2.9	n.s.
Missing data	6		3			
No anxiety	28	40.0	46	46.5		
Possible anxiety	11	15.7	21	21.2		
Probable anxiety	25	35.7	29	29.3		
HADS Depression					3.9	n.s.
Missing data	6		6			
No depression	32	45.7	55	55.6		
Possible depression	12	17.1	18	18.2		
Probable depression	20	28.6	20	20.2		

CH, Chronic headache, pMOH, Probable medication overuse headache

## Discussion

For the first time this cross-sectional, prospective, multicentre study provides comprehensive data from eight Austrian headache centres. Of 441 patients included 57 % had EH, 38 % had CH and 5 % did not give their headache frequency. The prevalence of migraine, TTH and pMOH was 49 %, 6 % and 16 %, respectively. In the previous month, the vast majority—more than 90 % – of the patients had used acute medication. The percentage was higher in EH than in CH. The proportion of patients who had used prophylactic drugs in the previous month was only 34 % and there was no difference between EH and CH. Even the proportion of patients who ever-before had used at least one of five standard prophylactic drugs, i.e. betablockers, flunarizine, topiramate, valproic acid and amitriptyline was not higher than 52 %. During the previous year two thirds of the patients had undergone MRI or CT and one out of seven attended an emergency department because of headache. Health related quality of life was poor or very poor in 12 % of the patients, more than 20 % indicated that their headaches had a negative impact on their earnings, one third experienced a negative impact on their career. The burden of headache in terms of lost days because of headache was severe in 45 % with more than 20 lost days in the preceding three months, and only half of the patients had noticed that their colleagues at work accepted their headaches. Symptoms of anxiety and depression, present in 45 % and 30 %, respectively, were significantly more present in CH than in EH.

### Headache chronicity and diagnoses

The greater proportion of males among patients with CH compared to EH should not only be seen as a selection bias, but might suggest that males are less likely to seeking help or being referred to a headache centre, if they have less frequent headaches. An epidemiological survey showed that more females than males with headache consulted their general practitioner [23]. With respect to the diagnostic spectrum, the majority of the patients with EH had migraine or probable migraine, and the vast majority of the patients with CH with or without pMOH was suffering from migrainous headache, comparable to previously published studies [12]. The percentage of patients with pMOH was 38–65 % smaller than in other studies [10, 11]. This may be due to different health systems in general, to different referral systems and to different study designs. In contrast to Austria, the Danish headache centre is the only tertiary headache centre in Denmark for 5 million inhabitants [10]. Referral regulations are less restrictive in Austria. Although patients should be referred to headache centres by neurologists, a substantial number of patients attends specialized headache centres on self-referral or on referral by their general practitioner. Bigal et al. [11] included patients with transformed migraine only in their study. Unexpectedly, the number of patients classified as CH without pMOH was high (99 of 169 patients). This may be caused by the cross-sectional study design.

The concordance between clinical diagnoses and Eurolight diagnoses was good for EH and moderate for CH. Conflicting diagnoses were associated with Eurolight diagnoses of probable migraine, probable TTH and other headaches. In clinical practice, neurologists tended to diagnose definite migraine or TTH more often. The Eurolight diagnosis of pMOH corresponded with the clinical diagnosis of MOH in only 52 %. The main reason for this discrepancy may be, that the Eurolight questionnaire covers the preceding four weeks, whereas clinically MOH was diagnosed, if the patients had overused acute medications for at least three months (according to ICHD-2). Moreover, some patients’ history did not indicate a clear-cut overuse. Two patients had cluster headache and were not considered as having MOH during their cluster episode despite their frequent use of triptans.

### Medication

The smaller percentage of patients with CH compared to those with EH who had used acute medication during the previous month may be explained by differences in the proportion of TTH or TTH-like headache, which was 11 % in EH, 16 % in CH and 24 % in CH without pMOH.

Patients with CH had ever used prophylactic drugs significantly more often than patients with EH. This was driven by two drugs: ever-before use of amitriptyline and valproic acid was more common in CH. In general, the proportion of patients using any prophylactic medication was comparable to other patient populations [11, 12]. Seen from the aspect that 38 % of the patients had CH, and only 39 % of them had used prophylactic medication during the previous month and 52 % had ever used one of five standard prophylactic drugs, patients with CH are undertreated. Since many patients taking part in the study were seen at the tertiary care centres for the first time, this reflects insufficient treatment in primary care and in neurological offices.

### Use of health care services

During the previous year patients with CH consulted general practitioners and emergency departments significantly more often than those with EH. Similarly, a population-based study from the USA [24] showed that the consultation of most health care professionals is numerically more common in chronic than in episodic migraine. Formal tests for statistically significant differences were not performed in this study [24].

The frequent use of neuroimaging as demonstrated in our sample is also reflected in a recent study from the USA [25]. This study covered more than 50 million patient visits with an ICD-9 diagnosis of headache and showed utilization of MRI or CT in 12.8 % of all visits for any headaches, in 10.2 % of the visits for headaches without red flags and in 9.8 % of all visits for any migraine. Even though our findings and the results of the US study cannot be compared because of different methods, both studies indicate overuse of MRI and CT in primary headaches. The emphasis on neuroimaging is also reflected by comparing the percentage of patients who had an MRI and/or CT during the previous year, i.e. 65.7 %, to that who had currently taken prophylactic medication, i.e. 34 %. The overuse of imaging and the underuse of prophylactic medication require further investigations to elucidate the role of the physicians and of the patients in this issue. On the one hand physicians may overuse imaging to defend themselves from the risk of misdiagnosis. On the other hand, daily clinical experience is such, that many patients with a long-lasting headache history want another MRI after some years. Sometimes these patients seem to be dissatisfied with their treatment or they fear a malignant disease, and it may be that in busy offices the easiest way to deal with this is to write a referral. It also points to the necessity of more continuing education for general practitioners and neurologists in the field of headache.

### Burden of headache

The burden of headache differs not only between patients with EH and CH, with the latter being affected more severely, but there seems to be also a relation to the type of the questions or questionnaires. Sixty-two percent of the patients reported at least 11 lost days during the preceding three months because of headache. In contrast, quality of life assessed by means of WHOQoL-8 was good or very good in 66 % and poor or very poor in 12 % only.

It was demonstrated that chronic migraine imposes a greater burden on the individual patient than episodic migraine in terms of lower quality of life and headache impact [12, 26, 27], and that patients with chronic migraine or pMOH had more workdays lost, more days with reduced productivity and more days lost for family or leisure activities than patients with episodic migraine [5, 12]. A recent study on headache yesterday in Russia showed that headache yesterday reduced productivity in 70 % of the patients [28]; likewise in an African cohort 68 % of patients with headache lost workdays [29]. Data on TTH are sparse, but a recent study found similar results for patients with chronic TTH [30]. In a longitudinal study patients with chronic migraine had lower household incomes and worked part-time more often than patients with episodic migraine [7]. Also, in our study a substantial number of patients experienced a negative impact on earnings and careers and they missed acceptance of their headaches at work.

### Psychiatric comorbidity

Psychiatric comorbidity, in particular depression and anxiety, has been studied extensively in patients with recurrent or chronic headaches [31]. The proportion of patients with evidence of anxiety and depression in our study was comparable to previous studies [31]. Similarly, our findings agree with the literature regarding a higher prevalence of depression and anxiety in patients with CH compared to those with EH [7, 32]. Patients with psychiatric comorbidity are at higher risk for chronic migraine and MOH [31]. Future studies should (further) clarify the impact of anxiety and depression on the use of healthcare services, treatment and burden of headache.

### Strengths and limitations

This was the first study in Austria investigating the prevalence, management and burden of EH and CH in specialized headache outpatient centres. Strengths are that all important headache centres participated in the study, the high participation rate among patients, and that we assessed clinical diagnoses in addition to Eurolight diagnoses. Possible limitations are that the Eurolight questionnaire does not allow to differentiate CH, and that the additional questionnaire on prophylactic medications was based on retrospective assessments and therefore may suffer from recall bias.

## Conclusion

In conclusion we provided detailed data on the prevalence, management and impact of headache in eight Austrian headache centres. A substantial number of patients has CH, including pMOH, and these patients utilize health care facilities more often and suffer from a greater impact of headache on their daily lives. Future studies should elucidate the reasons for the overuse of neuroimaging and the underuse of pharmacoprophylaxis as well as the impact of anxiety and depression on the management of recurrent or chronic headaches. Finally, our future efforts must aim at reducing the burden of headache by reducing imaging and improving pharmacological treatment.
